# Landmark-Based Updating of the Head Direction System by Retrosplenial Cortex: A Computational Model

**DOI:** 10.3389/fncel.2018.00191

**Published:** 2018-07-13

**Authors:** Hector J. I. Page, Kate J. Jeffery

**Affiliations:** Institute of Behavioural Neuroscience, Department of Experimental Psychology, University College London, London, United Kingdom

**Keywords:** navigation, head direction cells, landmark processing, retrosplenial cortex, attractor dynamics, vision, hippocampus

## Abstract

Maintaining a sense of direction is fundamental to navigation, and is achieved in the brain by a network of head direction (HD) cells, which update their signal using stable environmental landmarks. How landmarks are detected and their stability determined is still unknown. Recently we reported a new class of cells (Jacob et al., 2017), the bidirectional cells, in a brain region called retrosplenial cortex (RSC) which relays environmental sensory information to the HD system. A subset of these cells, between-compartment (BC) cells, are directionally tuned (like ordinary HD cells) but follow environmental cues in preference to the global HD signal, resulting in opposing (i.e., bidirectional) tuning curves in opposed environments. Another subset, within-compartment (WC) cells, unexpectedly expressed bidirectional tuning curves in each one of the opposed compartments. Both BC and WC cells lost directional tuning in an open field, unlike HD cells. Two questions arise from this discovery: (i) how do these cells acquire their unusual response properties, and (ii) what are they for? We propose that bidirectional cells reflect a two-way interaction between local direction, as indicated by the visual environment, and global direction as signaled by the HD system. We suggest that BC cells receive strong inputs from visual cues, while WC cells additionally receive modifiable inputs from HD cells which, due to Hebbian coactivation of visual inputs plus two opposing sets of HD inputs, acquire the ability to fire in both directions. A neural network model instantiating this hypothesis is presented, which indeed forms both BC and WC bidirectional cells with properties similar to those seen experimentally. We then demonstrate how tuning specificity degrades when WC/BC cells are exposed to multiple directionalities, replicating the observed loss of WC and BC directional tuning in the open field. We suggest that the function of these neurons is to assess the stability of environmental landmarks, thereby determining their utility as reference points by which to set the HD sense of direction. This role could extend to the ability of the HD system to prefer distal over proximal landmarks, and to correct for parallax errors.

## Introduction

The brain’s construction of an internal “map” of external space involves transformation from basic sensory inputs to higher-order representations, resulting in abstract concepts such as “place,” or “heading.” The study of spatial cognition can therefore help us to understand how the brain forms such concepts, which is broadly relevant to cognition more generally. Maintaining a sense of direction is a key aspect of navigation for any organism. Head direction (HD) cells signal current directional heading, representing a neural correlate for the sense of direction (Taube et al., 1990a; Taube, 2007). Here, we investigate potential mechanisms by which HD cells can learn about the visual world, using computational modeling based on recent experimental findings.

The experiment was conducted as part of a research programme aiming to investigate how sensory (mainly visual) inputs are transformed between primary cortex and the HD system. Using sensory inputs to update the sense of direction is a difficult problem for the brain to solve, because the incoming information is so dynamic, due to the animal’s movements. HD cells are principally updated by self-motion information regarding head rotations, a process known as path integration (McNaughton et al., 2006). However, the HD signal needs to be held stable relative to the external environment (Taube et al., 1990b; Goodridge and Taube, 1995; Goodridge et al., 1998). For this to happen, environmental landmarks must be assessed for stability. This necessitates an evaluation of newly-encountered landmarks relative to an internal sense of direction, so that only landmarks that have been learned as a stable source of directional information will update HD cell firing (Knierim et al., 1995; Knight et al., 2013). There is thus a chicken-and-egg problem—stable landmarks are used to orient the sense of direction, but the sense of direction is needed to determine whether landmarks are stable. Another issue is that sensory input is initially represented relative to the body (egocentric), while an internal sense of direction is relative to the external world (allocentric; Bicanski and Burgess, 2016). Use of environmental information to set the sense of direction equates to a transformation between egocentric and allocentric spatial reference frames (Vann et al., 2009; Knight and Hayman, 2014).

The experiment that motivated the present modeling study investigated the transformation from visual signals to the HD signal by recording from neurons in the waystations between visual and HD areas. Visual information is conveyed to the HD system via two cortical areas, postsubiculum (PoS) and retrosplenial cortex (RSC), both of which contain HD cells with similar properties (Taube et al., 1990a; Chen et al., 1994; Lozano et al., 2017). We focus here on RSC, which receives direct input from the visual cortex (Sugar et al., 2011). Lesions of RSC disrupt updating of HD cell firing following visual landmark rotations (Goodridge and Taube, 1997; Clark et al., 2010), and neuroimaging studies in humans suggest that it may be involved in the processing of landmark permanence (Auger et al., 2012; see Mitchell et al., 2018).

In recent work (Jacob et al., 2017) in which we explored cell responses in RSC, we gained some new insights as to how environmental inputs might inform a sense of direction. Rats were placed in a two-compartment apparatus in which compartments were, visually, 180° rotations of one another, so that the visual cues signaled one orientation in one compartment and the opposite orientation in the other compartment. The apparatus was situated in a curtained-off circular arena and rotated from trial to trial, to prevent use of distant directional cues such as traffic infrasound. To break the symmetry and allow the rat to orient itself, the compartments were scented differently, one with lemon and one with vanilla.

The original question was whether HD cells could indeed use these olfactory cues to break the visual symmetry of the space, but we found something rather unexpected. Traditional HD cells were found to maintain the same overall direction of firing between the two compartments, thus firing in one direction relative to the visual panorama in one compartment, and the opposite direction (i.e., the same direction in global space) in the other. The cells were able to retrieve the correct orientations even when the animal was randomly started in the lemon or vanilla compartment, thus answering the original question of whether the cells could use olfactory cues to break the visual symmetry. However, a population of RSC cells, in the dysgranular region, were found to switch directions by 180°, meaning that they rotated their firing directions when the rat stepped through the central doorway in the wall separating the compartments. We refer to these as between-compartment (BC) cells, because their bidirectionality was a function of exploration of the two-compartment space (that is, tuning curves in each sub-compartment were singular). These cells were co-recorded with the ordinary HD cells reporting global heading, which did not therefore rotate—this dissociation in itself was surprising since previously, directionally tuned cells had always been found to be concordant (Peyrache et al., 2015). Even more surprising however, was the discovery of a further population of cells that maintained two firing directions at 180° within each single sub-compartment. We refer to these as within-compartment (WC) cells. Such dual tuning curves in a single compartment have not been reported in any previous studies of RSC HD neurons and so we felt this phenomenon must have something to do with the overall rotational symmetry of the two-compartment space—but why, then, would it manifest in a single compartment?

Experience-dependent plasticity provides the only plausible explanation, since the cells must have acquired their dual tuning curves as a function of the rat’s previous exploration of the two-compartment space. We thus proposed that the bidirectional cells in Jacob et al. (2017) arose from an interaction between visually-driven and internally-maintained signals, shaped by Hebbian plasticity between the neurons.

In the present study, we devised a model to test this hypothesis. In our model, some RSC cells receive a strong input from the main HD network and thus are classic HD cells; another population receive strong visual inputs and thus become BC cells, and a third subpopulation receive inputs from both the HD signal and the visual inputs and thus become WC cells. These inputs are modified by Hebbian plasticity such that co-activation strengthens them, and uncorrelated activity weakens them. We demonstrate that this network will, in a rotationally symmetrical environment, come to form the three classes of response types (global tuning curve, local tuning curve and dual tuning curve) seen experimentally. We then propose a function for this network that explains the function of these three cell classes: namely, that bidirectional cells reflect a RSC role in both evaluating the usefulness of environmental sensory inputs for indicating current direction, and for incorporating direction as indicated by these inputs with a sense of direction maintained by the HD system. Thus, we suggest that these neurons use the HD signal as a benchmark against which to evaluate the stability of landmarks, and use stable landmarks to reset a drifted HD signal. They thus reflect a two-way interaction between incoming perception and the system’s current state, which may be a subset of a more general function for RSC in memory.

## Materials and Methods

### Key Features of Bidirectional Cells

**Figure 1:**
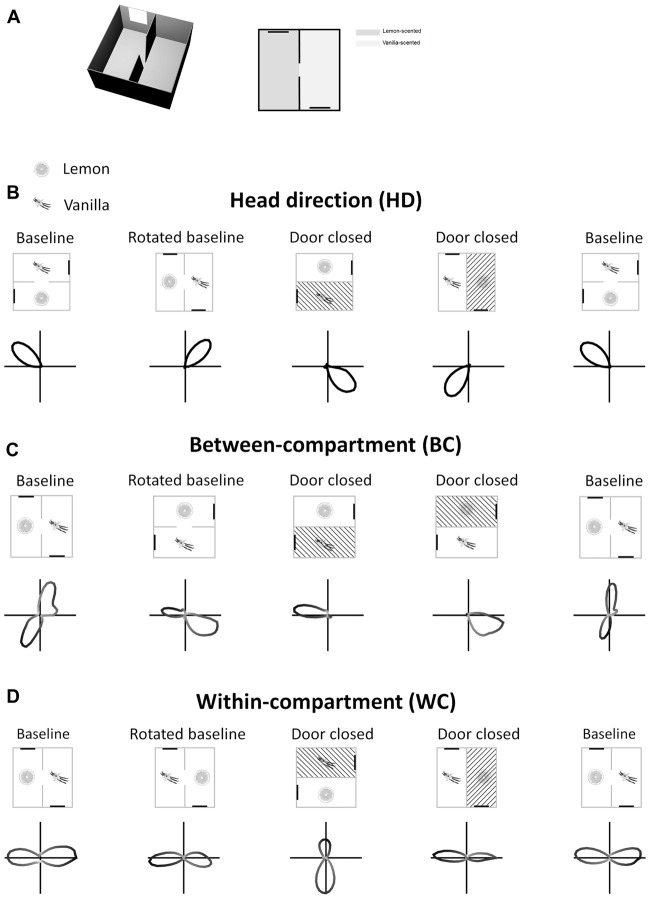
Tuning curves of three types of retrosplenial directionally tuned neuron: head direction cell **(B)**, between-compartment cell **(C)** and within-compartment cell **(D)**. Rectangles represent the recording apparatus, with thick black lines being cue cards used as visual landmarks. Shaded compartments are not accessible to the animal. Lemon and vanilla symbols denote the separate odors of the two compartments. Polar plots show firing rate of each cell types as a function of azimuth head angle. HD cells **(A)** have a single unimodal tuning curve across the apparatus as a whole, with preferred firing direction (PFD) remaining the same across compartments. BC bidirectional cells **(C)** have a bimodal tuning curve in the apparatus as a whole, but in a single compartment show a unimodal tuning curve which “flips” 180°. WC bidirectional cells **(D)** show bimodal tuning curves in both the apparatus as a whole and in individual compartments, with a dominant peak that “flips” 180°. All cell types rotate their tuning curves concordantly with apparatus rotations.

The model aimed to generate bidirectional cells having the properties seen by Jacob et al. (2017). These cells are shown in Figure 1. At a single-cell level, a sense of direction is expressed by a HD cell’s preferred firing direction (PFD). When the head is facing the PFD a HD cell fires at its maximum rate, and firing rate decreases progressively as the head moves away from the PFD. This results in a Gaussian tuning curve in HD space. An example of this, expressed as a polar plot, can be seen in Figure 1A (baseline condition). With this in mind, important points for the model to replicate are:

1. A globally stable HD cell PFD across the two compartments.
2. Rotation of HD cell PFDs with rotation of the apparatus.
3. A population of BC bidirectional cells, the PFD of which rotates (“flips”) 180° when moving BCs, but which is unidirectional within a single compartment.
4. A population of cells that have a bidirectional tuning curve within each sub-compartment (WC cells).
5. Unbalanced strength of tuning curve poles for WC cells, with the dominant/stronger pole having a higher firing rate than the non-dominant/weaker pole.
6. WC cell tuning curve dominant pole flipping 180°.
7. Persistence of all bidirectional cell firing in the dark.
8. Loss of bidirectional cell tuning when removed from the apparatus and placed in an open field.

### How RSC Processes Directional Information: A Hypothesis

**Figure 2:**
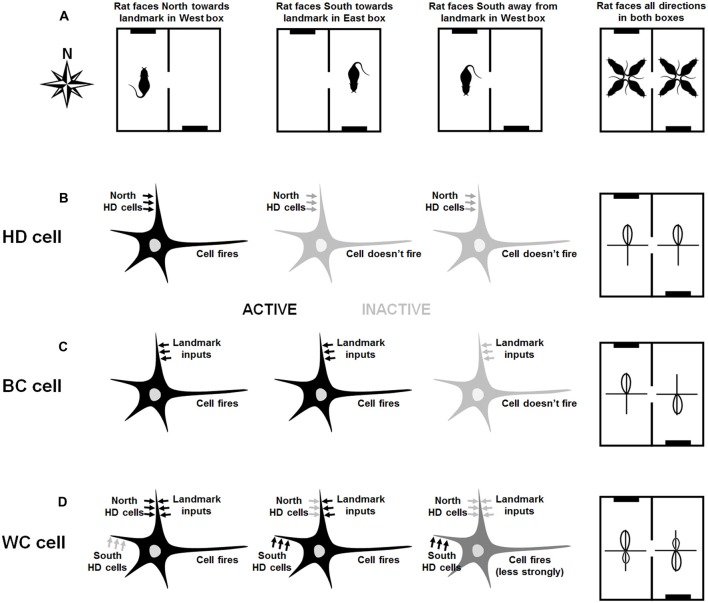
Hypothesized mechanism of directional cell firing characteristics in retrosplenial cortex (RSC), as proposed by Jacob et al. (2017). The figure shows various scenarios (left three columns) and the resultant overall tuning curves of the cells (rightmost column). Connectivity of the neurons is shown by the presence of arrows, and the current drive of each neuron in each scenario is shown by the shade of the arrows. Left-column plots: in the West compartment **(A)**, the rat faces the cue card (black line in the schematic). A hypothetical HD cell **(B)** is driven by the global HD signal and so fires when the rat faces North. A hypothetical BC cell **(C)** is driven by visual inputs and thus fires when the rat faces the cue card landmark. A hypothetical WC cell **(D)** receives inputs from both visual inputs and HD cells. It thus fires strongly when the rat faces the cue card, which lies to the North. Due to Hebbian co-activation with the landmark inputs, the inputs from the North HD cells increase in strength. Second-column plots: In the East compartment **(A)**, the rat again faces the cue card, which now is located in the South. The North HD cells are not firing and so the HD cell **(B)** does not fire. However, the BC cell **(C)** receives active landmark inputs, since the rat is facing the landmark, and so fires. The WC cell, which is connected to both landmarks and HD cells, receives active inputs from South HD cells as well as the landmarks: it thus fires strongly. Third-column plots: In the West compartment, the rat now faces away from the cue card **(A)**. The North HD cells are not firing and so the HD cell **(B)** does not fire. The BC cell does not receive active landmark inputs, since the rat is facing away from the landmark, so it does not fire either. The WC cell, which is connected to both landmarks and HD cells, receives active inputs from South HD cells as well as the landmarks: it thus fires strongly. Fourth-column plots: The overall activity of each cell is determined for all facing directions of the rat in both compartments **(A)**. The HD cell in **(B)** has a tuning curve that points North in both compartments. The BC cell in **(C)** has a tuning curve that reverse direction because it is controlled by the landmark, and the WC cell in **(D)** has a bidirectional tuning curve in both compartments, although it is slightly stronger in the direction aligned with the landmark.

Figure 2 shows the hypothesized bidirectional cell formation mechanism, based on varying responsiveness to internal and environmental directionalities.

BC cells are driven exclusively by visual inputs, although they project back to HD cell areas via RSC HD cells. In the West compartment (Figure 2A, left) a specific BC cell (Figure 2B) is driven by input from a cell that signals direction as conveyed by the local spatial environment, referred to in this article as a VIS (“visual”) cell. While these cells likely respond to several sensory modalities indicating the external environment, vision is the modality of interest in this case. We note that cells with the relevant properties, namely responsiveness to a visual cue located at a specific egocentric bearing to the animal, have been reported in posterior parietal cortex (Wilber et al., 2014), which has connections with RSC (albeit sparse ones; Olsen et al., 2017).

In the East compartment (Figure 2A, middle), the BC cell is driven by the VIS cell responding to the direction of the cue card, which is to the North. As a result of Hebbian coactivity, the connection from the BC cell to the RSC HD cell signaling North is strengthened. In the East compartment (Figure 2A, middle), the same BC cell (Figure 2B, middle) is driven by the same VIS cell, but this activity correlates now with the firing of the RSC HD cell signaling South. As a result, the connection from the BC cell to the RSC HD cell signaling South is strengthened. Following learning in both compartments, BC cells are driven by visual inputs and will therefore be unimodal in each compartment, but will rotate tuning curves BCs in line with the apparent rotation of visual landmarks BCs, and will thus appear bimodal in the apparatus as a whole. Each BC cell will project back to two HD locations, at 180° separation. In either compartment when facing away from the landmark (Figure 2A, right), this BC cell will not fire (Figure 2B, right).

WC cells are driven by both HD and VIS inputs, and also project back to the RSC HD cell layer. Consider a single WC cell (Figure 2C), which initially receives inputs from a particular VIS cell that is sensitive to the cue card landmark and thus fires when the rat faces it. In the West compartment (Figure 2A, left), the WC cell is driven to fire when the agent is facing the cue card, which in this compartment also corresponds to when the agent is facing North. The conjunction of pre- and post-synaptic firing results in Hebbian plasticity, strengthening the connection from the RSC HD cell with a PFD of North to the WC cell (we also assume the reverse WC-HD plasticity as well, though this is not essential to the model).

In the East compartment (Figure 2A, middle), the WC cell (Figure 2C) is still driven by VIS cell firing in the direction of the landmark. However, due to the apparent 180° rotation of the VIS BCs, this firing is now correlated with the firing of the RSC HD cell with a PFD of South. This correlation again results in Hebbian strengthening, this time of connections in both directions between the RSC HD firing to South and the active WC cell. When facing away from the landmark in either compartment (Figure 2A, right) the WC cell will still be active but will fire at a lower rate as it is driven by previously-learned HD connections alone (Figure 2C, right).

Following learning, each WC cell receives maximally from a specific VIS location, and the two HDs associated with that VIS in either compartment. Thus, a given WC cell will fire due to VIS plus HD in one global direction, and HD alone in the other global direction, yielding a bidirectional response profile with asymmetric peaks. This asymmetry will flip BCs, as the VIS will be 180° rotated. As a result, a given WC cell is bidirectional in each compartment, as well as in the apparatus as a whole.

The above-detailed mechanisms demonstrate the fact that RSC cell bidirectional firing is a reflection of the ambiguity of the VIS representation, relative to a global sense of direction corresponding to the internal representations of HD. In this model, therefore, it is considered that the main function of bidirectional cells is to perform a sort of bootstrapping operation: direction as indicated by environmental landmarks is processed in relation to an internal sense of direction, and then stable landmarks (i.e., those who have a consistent relationship to internal HD) are used to update the internal sense of direction reflected by the firing of HD cells.

HD cells are not explicitly generated in this model, but at the RSC level inherit their properties from other HD cell regions, which we labeled as anterodorsal thalamic nuclei (ADN; although in principle there are other possible candidates for the origin of this HD signal, e.g., postsubiculum or anteroventral thalamic nuclei). They rotate with apparatus rotations due to their anchoring to visual landmark cues, but are able to use the olfactory cues to conditionally regulate this anchoring, such that they fire in one direction relative to the lemon (“West”) cue set and the other relative to the vanilla (“East”) cue set. They are unipolar across all compartments, due to the ambiguous inputs they receive from WC/BC cells. In each compartment, RSC HD cells will receive conflicting inputs, separated by 180°, from WC/BC cells. Given that some of this input will be closer to the current RSC HD activity, supported by self-motion inputs, irrelevant input will be ignored by the RSC HD cell layer.

### Model Description and Simulation

**Figure 3:**
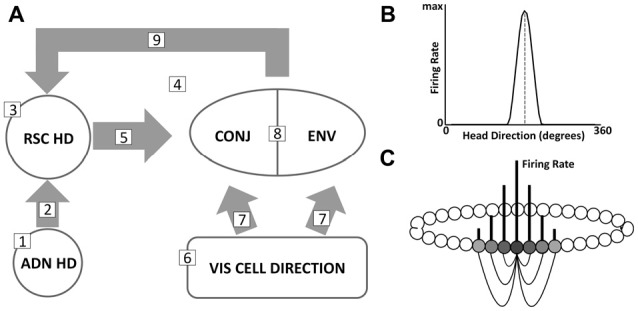
**(A)** Model schematic, detailing key regions and connectivity. Numbered points correspond to key model features listed in the text. **(B)** Example tuning curve of a single HD cell, with PFD marked (dashed gray line). **(C)** Stylized schematic of a continuous attractor network. A subset of connectivity (black lines) is shown for currently active cells, with gray vertical lines representing firing rate. Cells are arranged in a ring based on similarity of PFD, here reflected from dark to light gray relative to the most active cell (darkest). Connection strengths are also based on similarity of PFDs.

This section gives a short-form outline of key aspects of the model and simulations. This is supported by further precise details of model equations within the Supplementary Material. A model schematic is pictured in Figure 3A. The most important details are numbered in the diagram as follows:

1. A central ring of RSC HD cells receives input signaling current HD, from a continuous attractor ring of cells in the ADN. Such attractor architecture reflects the dominant model of HD cells (Skaggs et al., 1995; Zhang, 1996). Individual cells have a Gaussian tuning curve (Figure 3B) of PFD in HD space. Every cell is connected to every other cell with a strength that is a Gaussian function of the difference in pre- and post-synaptic PFDs. This arrangement is pictured with individual cells ordered by preferred direction (Figure 3C) but no such physical arrangement is required, provided connectivity is based on PFD. Given some inhibition, firing will take the form of a bump or packet of activity, representing current estimated HD. Whilst a distributed circuit between lateral mammillary nuclei and dorsal tegmental nuclei (LMN/DTN) is thought to constitute the core generative mechanism of the HD cells, the signal is conveyed via ADN, and this model simplifies the origin of ascending HD signal input.
2. ADN to RSC connectivity is pre-wired, with each RSC HD cell receiving connections of a strength based on the similarity of ADN HD and RSC HD PFDs.
3. The RSC HD cell layer does not contain recurrent collateral connectivity, although local inhibition is present.
4. This ring of RSC HD cells projects to a second layer of RSC cells, the bidirectional cells. The bidirectional layer can be divided into two subpopulations of cells firing to conjunctions of HD and VIS inputs (CONJ cells) and cells receiving VIS inputs alone (ENV cells). It should be noted that this distinction is on the basis of receiving HD connectivity: ENV cells do not receive HD inputs. This situation, with cells either receiving HD input or not, could be achieved *in vivo* by a single layer of bidirectional cells with varying proportions of HD inputs and a firing threshold, such that some cells are not driven to firing by their sparser inputs. It is expected that, via the mechanism outlined in the hypothesis, CONJ cells will become WC bidirectional cells, and ENV cells will become BC bidirectional cells.
5. RSC HD to CONJ cell connectivity self-organizes during the course of simulation via Hebbian plasticity.
6. Both CONJ and ENV cells receive inputs from the local spatial environment (VIS) layer, whose activity reflects a multi-modal sensory representation of current allocentric direction. In all initial simulations, activity in this VIS ring reflects the facing direction of the rat in local visual space, as if they were receiving retinotopic inputs. Thus, unlike HD cells, VIS cells rotate their representation BCs; capturing the apparent rotation of the environment due to the rotational symmetry BCs. In simulations addressing the parallax problem (see: *“Bidirectional Cells Mediate Landmark Learning, Possibly Solving the Parallax Problem”)*, VIS cells are modified in response to be simple binary landmark detectors, firing at maximum rate when a given landmark falls within a 90° field of view and not firing otherwise.
7. VIS to bidirectional connectivity is pre-wired. Bidirectional cells are assigned a notional PFD within the VIS cell layer. VIS to bidirectional connections are then set, as with ADN to RSC HD connectivity, based on the similarity of presynaptic and postsynaptic preferred directions. The entire range of VIS directions is represented within both CONJ and VIS subpopulations.
8. Local inhibition is present with the CONJ/ENV cell layer.
9. Both populations of CONJ and ENV cells project back to the RSC HD layer, with connections that self-organize due to Hebbian plasticity.

**Figure 4:**
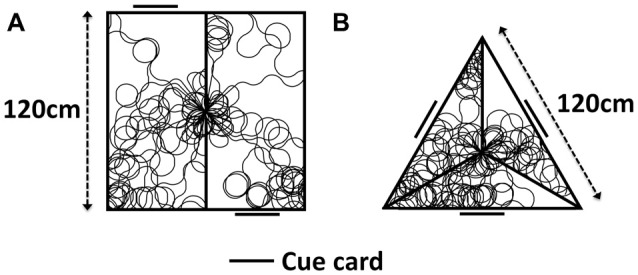
Apparatus used for model simulation, with sample simulated paths. **(A)** Two-compartment apparatus as used in Jacob et al. (2017). **(B)** Simulated triangular apparatus featuring 120° rotated compartments.

This model is first simulated in two separate environments, pictured in Figure 4. The first (4A) directly replicates the experiment of Jacob et al. (2017). A 120 cm × 120 cm box is divided into two rectangles 160 cm × 80 cm, with a single partition leaving a 10 cm door in the center. On one wall of each compartment is a single cue card, with this card being on opposite walls in different compartments. This results in the two compartments having identical VIS cell representations, rotated 180° from one another, with the direction indicated by the VIS cell firing aligned with the true global direction of the agent in one compartment, and rotated by 180° in the other.

The second of these environments (B) is an extension of empirical work, in order to illustrate the general nature of our hypothesis. It is an equilateral triangular apparatus, with each wall 120 cm in length. A partition extends towards the centroid from each corner, stopping 10 cm short of the centroid to leave a three-way door. A cue card is positioned on the wall opposite each apex. Each compartment appears visually identical. In one compartment, direction indicated by the VIS cell layer is aligned with the true global direction of the agent, whilst the VIS representation is rotated by 120° clockwise or counter-clockwise in each of the other two compartments. During simulation, the model is placed within either of the environments in Figure 4. A random walk is generated, using custom built MATLAB scripts, with the simulated agent moving through the environment and transitioning BCs. ADN activity is represented as a Gaussian input to the RSC layer, centered on the true HD of the agent. In one compartment, the VIS representation is set to be equal to the ADN direction, in others it is offset (either by 180° in the two compartment simulations, or by +/− 120° in the three compartment simulations). RSC HD to CONJ/ENV and CONJ/ENV to RSC HD weights are updated. For the last 30 s of simulation, weights are held constant in order to construct tuning curves of CONJ/ENV cells following system self-organization.

### Analysis of Model Performance

To analyze network performance at a single cell level, tuning curves are constructed by binning HD in 6° increments and then taking the mean firing rate for each bin. The tuning curve is then smoothed using a 5-bin moving average filter. Tuning curves are constructed in this manner for the final 30 s of simulation, for all compartments together and for each compartment individually. In order to avoid spurious effects of uneven sampling of all HDs and compartments, the final 30 s of simulation consists of the rat rotating on the spot at a constant speed of 36°/s, teleporting between a point in each compartment. This 30 s was split equally BCs (15 s each in the two-compartment apparatus, and 10 s each in the three-compartment apparatus).

To analyze model performance at the network level, tuning curves are autocorrelated. The number of peaks in this autocorrelation is taken to be the number of peaks in the tuning curve. Two-compartment simulations should produce cells with two tuning curve peaks at 180° separation, and three-compartment simulations will similarly produce three tuning curve peaks at 120° separation. This process also allows the tuning curves of the entire layer to be aligned and visualized.

Stability of network response is assessed by examining changes in PFD. Once a tuning curve is constructed, the PFD of a given cell is given by

(1) $$PFD_{i}=arctan\left(\frac{\sum_{i}r_{i}\sin(x_{i})}{\sum_{i}r_{i}\cos(x_{i})}\right)$$

where *x_i_* is the mean of tuning curve bin *i* with firing rate *r_i_* and *arcctan* denotes the quadrant-specific arctangent.

The spatial/temporal stability of this PFD can be recalculated over different periods of time, or space. Spatial stability of ADN cell PFDs is explored by calculating a tuning curve for each quadrant of a circular apparatus and then comparing the average per-quadrant tuning curves. Temporal stability of ADN cell PFDs is explored by calculating the PFD of each cell for different, successive, simulation time periods.

## Results

### Bidirectional Tuning Curves Emerge From Hebbian Synaptic Plasticity

**Figure 5:**
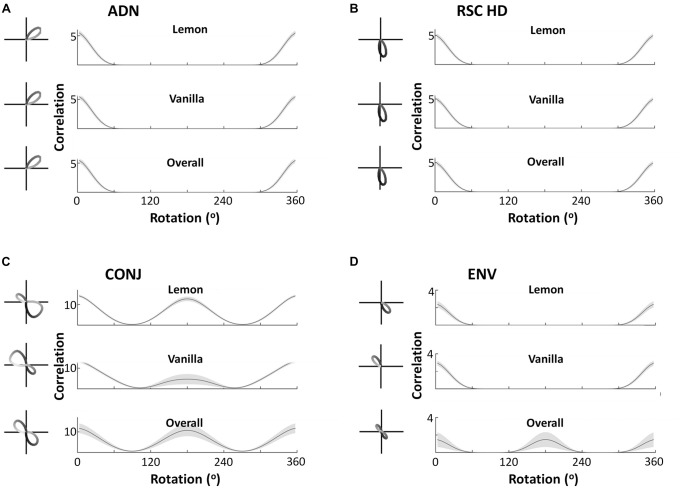
Cell responses during two-compartment simulations. Example tuning curves and autocorrelation plots are shown for the overall apparatus and for the two subcompartments, for four cell types—anterior thalamic head direction cells (ADN), restrosplenial head direction cells (RSC HD), conjunctive cells (CONJ) and environment cells (ENV). Autocorrelations pictured are the mean, with standard errors shown as shading, across all cells in the layer. This demonstrates that the key cell types observed by Jacob et al. (2017) are replicated in the model, at both a single cell and network-wide level. ADN, RSC and ENV cells are unidirectional across compartments, with PFD in ENV cells rotating BCs. This is reflected by ADN, HD and ENV tuning curves having a single autocorrelation peak in either compartment (**A–C**, top two plots), but only ENV cells being bidirectional in the apparatus as a whole as reflected by a two-peaked autocorrelation (**D**, bottom plot). CONJ cells, in contrast, are bidirectional WCs (**C**, top two plots) and in the apparatus as a whole (**C**, bottom two plots), as reflected by two-peaked autocorrelations in all cases.

Observation of tuning curves shows CONJ cells becoming WC bidirectional cells, and ENV cells becoming BC bidirectional cells. Figure 5 shows example tuning curves from self-organized CONJ (5C, left) and ENV (5D, left) conjunctive cells, as well as RSC (5B, left) and ADN (5A, left) HD cells, in each compartment and in the apparatus as a whole, after a simulated 600 s exploration in a two-compartment apparatus. CONJ cells demonstrate WC bidirectionality, with a dominant peak that swaps direction BCs as seen experimentally. In contrast, ENV cells have a unidirectional tuning curve in a single compartment, which swaps direction BCs, yielding bidirectional tuning in the apparatus as a whole. Again this is as seen experimentally. HD cells, whether ADN or RSC, always remain unidirectional with static PFD across compartments.

This analysis is repeated at a network level, to show that observations from example cells generalize to entire cell layers. Figure 5 also shows the mean tuning curve autocorrelation, across all cells of each type (5A–D, right). This depicts the correlation of a tuning curve with itself at different amounts of rotation, allowing easy observation number of tuning curve peaks, and their separations across all layers. This confirms that results observed from single cells extend to the entire layer of cells. In the apparatus as a whole all CONJ/ENV cells are bidirectional, with peak autocorrelation at 180°, and all HD cells are unidirectional. Splitting by compartment we see more specific responses, with CONJ/ENV cells differing. Peak autocorrelation is at 180° rotation for CONJ cells, even within single compartments, but ENV cell autocorrelations are not bimodal in individual compartments. The absolute value of the CONJ correlation is lower at 180° separation in single compartments, indicating the relative weakness of a second peak driven only by HD inputs.

**Figure 6:**
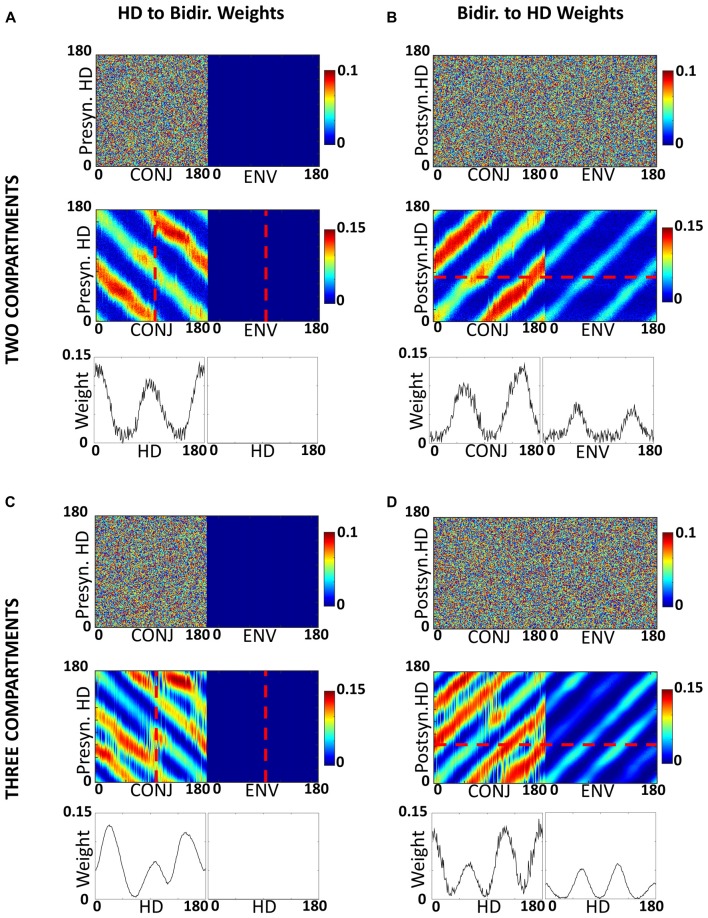
Development of inputs to CONJ and ENV cells. **(A)** RSC HD to CONJ/ENV layer weights for two-compartment simulation. Top shows initial weights coming from HD cells and projecting to a single postsynaptic CONJ (left) and ENV (right) cell, marked on bottom plot with red dashed lines, color-coded as shown in the color bar. These are randomly allocated for the CONJ cell and non-existent (zero) for the ENV cell. Middle shows post-learning weights, for the entire population. The CONJ cell receives HD inputs that are clustered around two directions 180° opposed; the ENV cell does not receive HD inputs. Bottom plots show final weights for the population; each CONJ cell receives, like the cell in the top panel (shown by the red dotted line), inputs clustered at 180°. **(B)** Equivalent plot to **(A)**, showing CONJ/ENV to RSC HD weights. **(C,D)** Equivalent plots to **(A,B)**, for three-compartment simulations.

The self-organized weight structure matches the hypothesized network. Observation of the weight structure (Figures 6A,B) indicates that weights to and from the bidirectional layer have self-organized in the expected manner, with 180° separated bimodal distributions. Note that, as per model assumptions, ENV cells do not receive RSC HD cell inputs, but both CONJ and ENV cells project in a specific bidirectional manner back onto the HD layer.

### Tridirectional Tuning Curves Are Predicted in a Three-Compartment Environment

**Figure 7:**
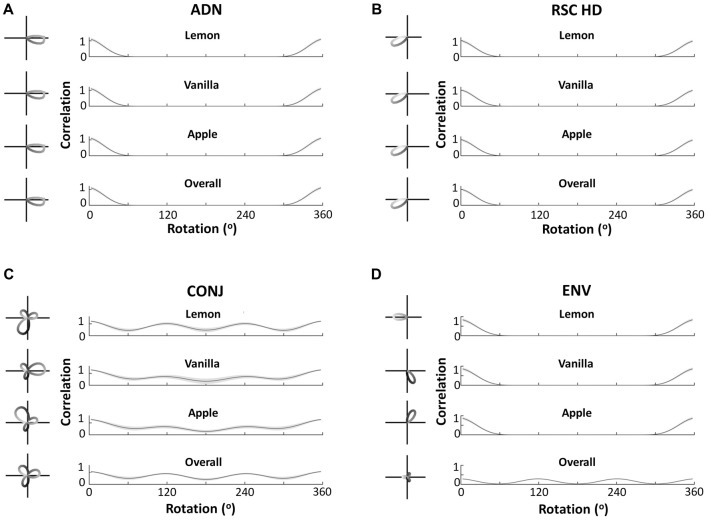
Cell responses during three-compartment simulations. Figure layout conventions and labeling as in Figure 5. This figure demonstrates equivalent findings to Figure 5, with bidirectionality in all forms becoming tridirectionality, due to the three-way rotational symmetry of the triangular apparatus.

Central to our hypothesis is the idea that bidirectionality is learned from the specific environment used by Jacob et al. (2017). Therefore, a three-compartment simulation should yield trimodal tuning curves: the simulation result confirms this intuition. Figure 7, similarly to Figure 5, shows example tuning curves and average autocorrelations across the entire layer, for each of the four cell subtypes, following simulation of 600 s in the three-compartment apparatus. Whilst ADN and RSC HD cells remain unidirectional and with the same PFDs, CONJ and ENV cells now have tridirectional tuning curves in the apparatus as a whole. Subdivision of tuning by compartments reveals that ENV cells are unidirectional with a PFD that rotates 120° BCs, and CONJ cells are tridirectional, with a dominant peak that also rotates 120° BCs.

Unlike CONJ cells in two-compartment simulations, and ENV cells in both simulations, CONJ cells in the three-compartment simulations did not uniformly develop tridirectional tuning curves. This is likely due to the added difficulty of learning three compartments, and it should be noted that a significant portion of CONJ cells were tridirectional in all compartments. This will be explored further in the section “*Development of Cell Response Properties*.”

Tridirectional tuning is supported by a specific organization of weights to and from the CONJ/ENV cell layer. Figures 6C,D show, similarly to Figures 6A,B, self-organized weight profiles to and from the conjunctive cell layer. Following simulation in a three-compartment apparatus, weights from HD to both conjunctive cell subtypes, and from CONJ back to HD, are trimodal.

### Development of Cell Response Properties

**Figure 8:**
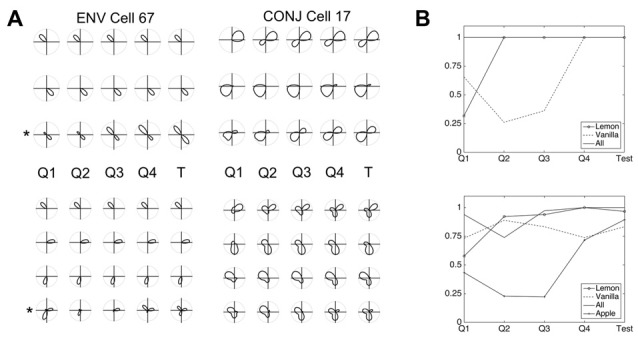
Development of CONJ and ENV firing properties. **(A)** Tuning curves for ENV cell 67 (left upper and left lower panels) and CONJ cell 17 (right upper and right lower panels) are shown for each quartile of simulation and for test phase (starred plots). **(B)** Proportion of cells in layer with mature tuning curves (bimodal in the two compartment simulations, top panel, and trimodal in the three compartment simulations, bottom panel) for the same time intervals as **(A)**.

We next looked at the development over time of both cell tuning curves and of weight profiles. Key multidirectional cell features appear to develop after a relatively short time. Figure 8A shows tuning curves of CONJ and ENV example cells taken every quarter-trial (150 s, plots labeled Q1–4) of training and during testing (plots labeled T), for both two-compartment (top two panels) and three-compartment (bottom two panels) simulations. Tuning curves for the same example cells are shown in each individual compartment, and in the apparatus as a whole (starred plots). For ENV cell 67, bidirectional/tridirectional tuning is visible in the apparatus as a whole from the first quartile of simulation, as these cells are simply responding to VIS inputs.

These plots indicate that different aspects of bidirectionality can emerge at different times. CONJ cell 17 is bidirectional within one of the compartments from Q2, and weakly in the other by Q4. In the apparatus as a whole however, BC bidirectionality is present from Q1. Again, this is due to the pre-wired nature of VIS to CONJ cell connectivity. Contrastingly, tridirectionality is a harder problem for the network to solve, and CONJ cell 17 shows WC tridirectionality in only two of three compartments. This suggests that a mixture of tridirectional and bidirectional cells could form in three-compartment experiments.

Looking at development of the network as a whole reinforces the idea that within- and BC multi-directionality arise at different times. Accompanying these single-cell examples are summary plots (Figure 8B), showing the proportion (0–1) of CONJ cells with fully formed tuning curves (i.e., bimodal, top, and trimodal, bottom) at the same time intervals. For two-compartment simulations it seems that within compartment bidirectionality occurs by Q2 in the lemon compartment, but that the vanilla compartment takes longer to learn. This suggests that learning in one compartment affects learning in another. For three-compartment simulations, CONJ cells struggle to develop tridirectionality in all compartments and show non-linear development, reinforcing this point.

**Figure 9:**
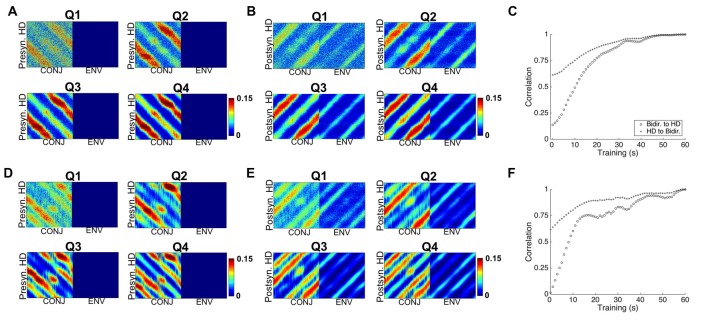
Development of weight profiles over time. **(A)** Snapshots at end of each quartile of two-compartment simulation for RSC HD to CONJ/ENV layer weights. **(B)** Equivalent plots for CONJ/ENV to RSC HD weights. **(C)** Correlation of both weight profiles with their final (Q4) state over the course of two-compartment simulation. **(D–F)** Equivalent plots to **(A–C)**, for three-compartment simulations. Whilst axes numbers are omitted for space reasons, numbers of HD, CONJ and ENV cells are as in Figure 5.

The idea of interference BCs can be investigated by looking at the development of weight profiles for the bidirectional cell layer. Figure 9 shows for two compartment simulations the HD to bidirectional (9A) and bidirectional to HD (9B) weights at each quartile of simulation for two-compartment (top) and three-compartment simulation (bottom, 9D,E). Also pictured are plots of the correlation of the weight profile with the final weight profile over the course of simulation for two-compartment (9C) and three-compartment (9F) simulations.

The bulk of learning happens relatively quickly. In both types of simulation, the weight profiles resemble their final profile by Q1, and Figures 9C,F tell us that at this stage (15 s) correlation between the weights and final is already close to 75%. Learning is not uniform for all compartments and HDs, with diagonal striped bands of weights being of varying weakness between one another as well as across their length. For example, moving for Q1 to Q2 for HD to CONJ weights (9A), we see that one set of bands, which corresponds to one relationship between HD and visual directionalities and therefore to one compartment, is developed more quickly.

In essence, learning in one compartment develops a connection between one presynaptic HD cell and a given CONJ cell, and a different CONJ cell in the other compartment. The final weight profile is a balance between the effects of each individual compartment. This antagonism BCs will be even more of a factor for the triangular apparatus, and is likely the cause of non-linear development of tuning curves as seen in Figure 8B.

### Theoretical Limits on Number of Compartments Represented Causes Loss of Tuning Specificity in the Open Field

If bi- and tri-directionality are products of the specific manner in which this RSC network mediates landmark influence on HD regions, then what limits the number of distinct relationships between VIS and HD that the network can represent? For a given WC cell, the cell will receive a Gaussian set of weights from a specific HD: one Gaussian profile of input for each compartment with a different relationship between VIS and HD activity. We can therefore consider a multi-directional tuning curve formed through experience of multiple VIS-to-HD relationships (i.e., multiple compartments) as the overlap of multiple Gaussian unidirectional curves. For example, the two-compartment apparatus used by Jacob et al. (2017) generates WC cells with a bimodal tuning curve comprising two Gaussian peaks, and the three-compartment apparatus similarly causes a trimodal tuning curve of three Gaussian peaks, but this system could in theory learn about more identical yet rotated compartments. The signal-to-noise ratio of a single cell’s response will thus depend on the number, width, and spacing of unidirectional curves, which is equivalent to the number and angular offset of compartments experienced.

**Figure 10:**
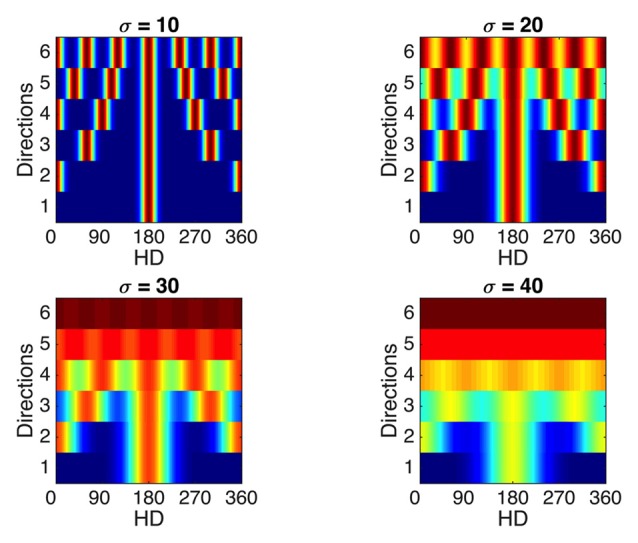
Effects of sensitivity to multiple compartments on tuning curve specificity. Considering a single WC cell, which experiences N evenly spaced rotated compartments, the final tuning curve will be formed by addition of N Gaussian curves evenly spaced through 360°. Tuning curves of a single WC cell responsive to multiple evenly spaced rotated compartments (y axes) are presented for a range of Gaussian curve widths (subplots). Firing rate values range from zero (blue) to the maximum value (dark red). As the number of compartments (N)—and equivalently, directions—represented by the WC cell increases, the tuning curve becomes less specific to a single direction, and this trend is exaggerated for broader width of individual Gaussians (sigma values for each subplot). This demonstrates that a theoretical limit exists on the resolution ability of this RSC network to represent multiple compartments.

To illustrate this, Figure 10 shows, for a variety of widths and with constant spacing, the tuning curves of a single cell with increasingly multiple directionality of response. This serves to illustrate the fact that with increasing width and increasing number of peaks, the cell’s response becomes increasingly less specific to a given direction. Thus, a limit exists in the capacity of the RSC network to reliably distinguish multiple visually indistinguishable compartments.

**Figure 11:**
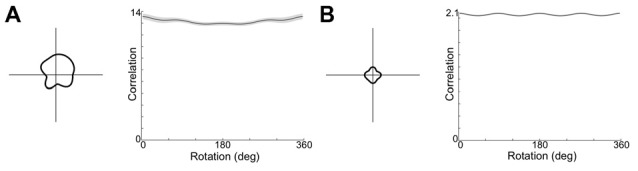
Open field simulation causes loss of tuning curve specificity. **(A)** Example single cell tuning curve (left) and mean tuning curve autocorrelation (right) for CONJ cells. **(B)** Equivalent plots for ENV cells.

It is possible that moving to a square open field arena, CONJ and ENV cells are responsive to the four-way geometrical symmetry. Given a broad enough spread of weights and inputs in the model, it could be that the loss of tuning results from learning a relationship between HD inputs and VIS inputs which rotate by 90° between quadrants. To test this, the model was re-run in a square apparatus, with the VIS representation rotating by 90° between quadrants and with a wider distribution of weights to and from the CONJ/ENV cell layer (*σ* = 40 up from 20). Figure 11 shows example resulting single cell tuning curves (left) and mean tuning curve autocorrelation across the entire cell layer (right) for both CONJ (11A) and ENV (11B) cell layers. Notably, specificity of cell tuning is lost, and this extends to the entire layer for both sorts of cells. Therefore, it is plausible that experimentally observed loss of tuning in a square arena is due to a RSC circuit attempting to process the four-way symmetrical geometry of the open field.

### Bidirectional Cells Mediate Landmark Learning, Possibly Solving the Parallax Problem

We consider here the possibility that BD cells might serve to correct parallax in the HD signal. Parallax is the theoretical distortion that should occur to landmark-driven HD signals, caused by the fact that nearby landmarks lie in a slightly different relative direction depending on where they are viewed from in the environment (Bicanski and Burgess, 2016). Our model of bidirectional cells provides a mechanistic account of how this error correction might occur, and in doing so it also explains why distal landmarks are preferred by the HD system.

**Figure 12:**
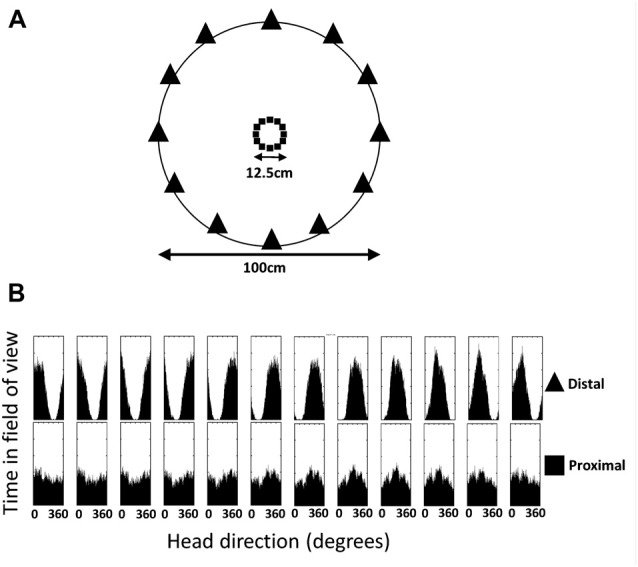
Distal and proximal landmarks occur in the field of view with differing probabilities relative to HD. **(A)** Environment used for simulations, showing proximal landmarks (small central squares) and distal landmarks (perimeter triangles). **(B)** Histogram of head directions at which each landmark is visible. Distal landmarks are clearly seen to generate peaked Gaussian probability distributions, while proximal landmarks do not.

To illustrate this capability, we simulated a random walk around a 1 m diameter circular environment that possessed an array of point-like landmarks arranged around the circumference (Figure 12A). In reality, we assume that the input to the network would be a panorama rather than an array of points, but experimental data on the exact details of visual input to the HD system are currently lacking. The symmetry and number of landmarks were chosen for convenience, but we have no reason to suppose that these parameters are critical to the functioning of the network. We chose 12 landmarks because a landmark-detecting cell with a visual field of 90°, which is the tuning width of a typical HD cell, would almost always be able to see at least one landmark. The VIS layer was thus modified to contain 12 cells acting as binary landmark detectors, firing at a rate of 1 when a landmark fell within a 90° field of view and of 0 otherwise.

The core intuition of this adjusted model is that, whilst a landmark may well be seen at multiple HDs, distal landmarks will be seen with a peaked (Gaussian) probability distribution centered on a specific HD. A Hebbian learning mechanism would thus form a peaked Gaussian set of connections from a landmark detector cell onto the HD layer. Multiple such landmarks, visible at any one time, combine their influence onto the HD layer to provide a probabilistic estimate of the current HD. In contrast, proximal objects will be seen at a broader range of HDs, yielding a less-peaked (and therefore less directionally-informative) set of connections. This is illustrated in Figure 12B, which shows a histogram of HDs at which a given landmark is seen for 12 proximal landmarks (squares) and 12 distal landmarks (triangles) during a random walk.

**Figure 13:**
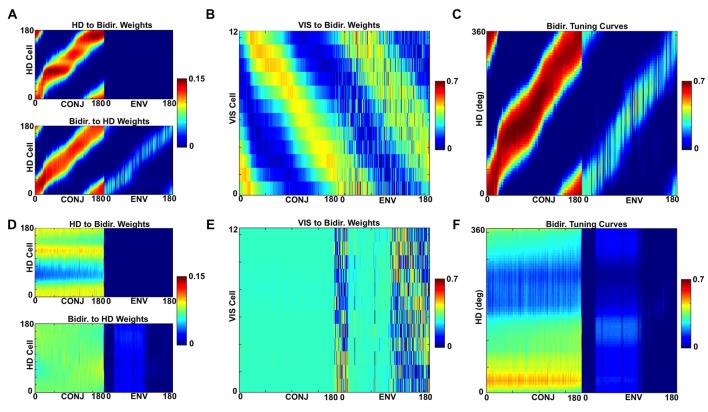
Differing probability relationships cause distal, but not proximal, landmarks to form a stable landmark processing network. **(A)** HD to bidirectional cell (top) and bidirectional to HD (bottom) weights for distal landmark simulations. **(B)** VIS to bidirectional weights for distal landmark simulations. **(C)** Bidirectional cell tuning curves in HD space for distal landmark simulations. **(D–F)** Equivalent plots for proximal landmark simulations.

Following simulation with a ring of distal landmarks, the statistics of landmark visibility combined with Hebbian learning have led to a specific pattern of connectivity from VIS to bidirectional cells (Figure 13B). CONJ cells form specific tuning curves in HD space (Figure 13C), and specific profiles of connectivity both to and from HD cells (Figure 13A). In contrast, simulations with a ring of proximal landmarks at 1/8 of the radius (Figures 13D–F) result in disrupted bidirectional cell connectivity to and from the HD cell layers, and non-specific bidirectional cell tuning.

**Figure 14:**
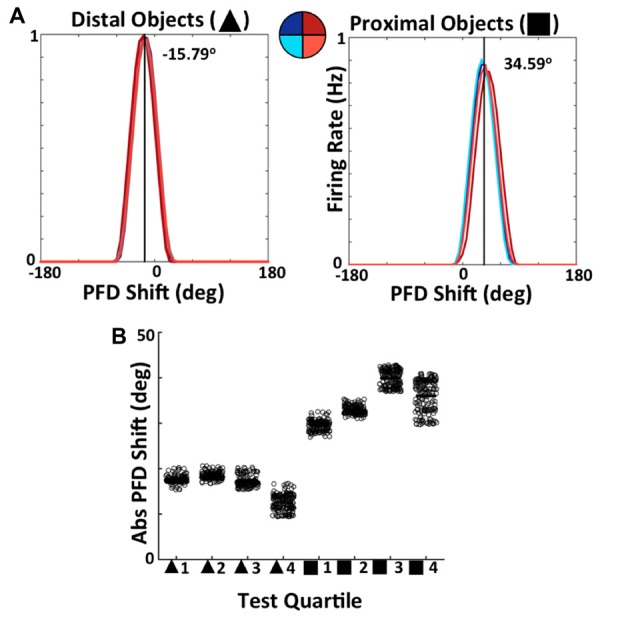
Simulated ADN HD cell tuning is stable across both space and time when the network uses distal, but not proximal, landmarks. **(A)** Comparison of average tuning curve, expressed as a shift from PFD of expected tuning curve, across quadrants of apparatus for distal landmark (left) and proximal landmark (right) simulations. **(B)** Tuning curve PFD, expressed relative to PFD of expected tuning curve, for each 30 s quartile of 120 s of simulation for distal and proximal landmark simulations.

Importantly, the differing resultant connectivity after simulating with proximal vs. distal landmarks has an effect on the stability of ADN HD cell tuning across both space and time. This is made clear by examining the drift in ADN HD cell tuning due to noisy path integration built into the model. During the final, additional, 120 s of simulation, all learning was turned off and ADN tuning curves were constructed for every 30 s individual, as well as for the entire 120 s for each quadrant. Figure 14A shows an equivalent analysis to that of Bicanski and Burgess (2016). During the final 120 s of simulation, tuning curves for each ADN cell have been constructed for each quadrant of the circular apparatus. These curves are then shifted by the amount each cell is expected to code for based on their arrangement at the beginning of simulation (i.e., prior to learning). The average shifted tuning curve for each quadrant is then plotted. When visual landmarks are distal (Figure 14A, left), tuning curves across quadrants align, indicating consistent ADN cell tuning within space. With proximal landmarks (Figure 14A, right) there is far more deviation across quadrants, indicating unstable ADN tuning across the apparatus. This is also reflected by the fact that the average deviation from expected tuning is larger for proximal landmark simulations (−34.59°) than for distal landmark simulations (−15.79°). These averages reflect drift of the HD system as a whole before VIS inputs have self-organized enough to stabilize PFDs.

Figure 14B shows the results of an equivalent analysis splitting the 120 s test phase in 30 s quartiles of time. For each individual ADN cell, the PFD every 30 s is compared to the PFD that the cell is expected to have based on the model arrangement prior to learning, with each point on the scatter plot representing a cell. This shift/drift is expressed as an absolute value. Note that distal landmark simulations result in ADN PFDs that drift less initially, as shown by the smaller drift in quartile 1, and stabilize over time as shown by the relatively smaller drift in PFDs across quartiles. This clearly demonstrates the ability of BD-to-HD cell connectivity formed via experience of distal landmarks, but not via experience of proximal landmarks, to stabilize HD cell PFDs across time.

## Discussion

The present study investigated at a theoretical level how the brain uses the visual environment to establish and update the sense of direction, as reflected in the activity of HD cells. We began with recent experimental findings that the directional coding of neurons in RSC is supported by three types of neurons showing differential responsiveness to landmarks (Jacob et al., 2017). Specifically, as well as HD cells expressing a global HD signal, this study also found BC cells having single tuning curves that rotated between one compartment and its connected, opposed counterpart, and WC cells that expressed two opposing tuning curves within each compartment. We hypothesized that the three cell types arise from variable coupling between visual inputs and HD cells, and simulated this proposal with a firing-rate model of a ring attractor. We demonstrated using this network that these two new types of directional tuning, BC and WC cells, indeed emerge. We then used the network to show that in environments with threefold or higher rotational symmetry directional tuning rapidly broke down, which may explain why it has not been observed previously in open field experiments. A slightly modified version of this model is shown to be able to use visual landmarks as stable reference points for the HD system. The model is shown to achieve this for distal, but not proximal, landmarks, suggesting a reason for the HD system’s affinity for distal landmarks. Below, we examine aspects of the experimentally observed phenomena that may be explained by the model, and propose a way to further test whether the model is a good description of the underlying circuitry. Finally, we offer some speculations about what this network might be for.

### Phenomena Explained by the Model

A curious observation from the Jacob et al.’s (2017) study was the existence of WC cells, expressing two opposing tuning curves even in a single compartment. Bipolar tuning curves have never previously been observed in RSC recordings (Cho and Sharp, 2001; Lozano et al., 2017) and so we hypothesized that they might result from experience of the unusual configuration of the two opposed compartments, which affected subsequent cell behavior in a single compartment. Furthermore, the bipolar tuning curves of WC cells were observed to be asymmetric, having one slightly larger and one slightly smaller peak, the orientation of which flipped by 180° by contrast with HD cells which did not rotate at all. In this sense they more resembled the BC cells, and indeed the BC cells could be considered an extreme version of WC cells in which the minor peak is non-existent.

Our model attempted to explain these observations in terms of feedback from HD cells. WC cells, which receive such feedback, are proposed to acquire inputs from two sets of HD cells during initial exploration of the two-compartment space, while BC cells, which receive little or no HD feedback, do not. By introducing such connectivity into the system and then allowing it to self-organize we did indeed see the emergence of bipolar tuning curves with a dominant, flipping peak. The reason for the asymmetry in the peaks is that in one of the two opposing directions the visual cues line up with the original HD, so the active HD cells combine with the visual cues to drive the cell strongly. In the other direction then the cell is being driven only by its acquired second set of HD cells, and fires more weakly.

Our model may also explain preliminary data from Jacob et al. (2017) suggesting that bidirectional cells, but not HD cells, lose their tuning curve specificity in open field recordings. We found that simulation of an apparatus with four-fold rotational symmetry generated cells with non-specific tuning curves, suggesting that perhaps the four-way symmetry of the square apparatus in the experimental study had crossed a threshold beyond which multiple peaks could not be supported.

### Predictions From and Extensions of the Model

As well as confirming the plausibility of our proposal about the formation of BC and WC cells, our model yields a number of predictions. Most obviously, recording in a compartment with threefold rotational symmetry should yield tridirectional cells, both within- and BC types. Relatedly, the hypothesis that bidirectionality is a product of the environment, rather than a fundamental characteristic of these cells, could also be tested by preventing synaptic weight updates *in vivo*, perhaps by infusion of an NMDA receptor blocker: we should see BC cells but not WC cells, as our model starts with organized VIS to CONJ/ENV weights, and self-organizes weights between CONJ and HD cells only. In reality, VIS to CONJ/ENV weights would self-organize in tandem with weights between HD and CONJ/ENV, and therefore the *in vivo* effects of blocking plasticity would be hard to predict from this model. It is likely that no directional preference would be observed: not even unimodality.

The CONJ cells in our model were the ones having combined sensory and HD inputs, which rapidly evolved to be WC cells. Our model predicts that CONJ cells would resemble HD cells in a single compartment apparatus, and become bidirectional when subsequently exposed to a different two-compartment apparatus. After experiencing the two-compartment apparatus, CONJ cell responses in a single compartment would likely depend on experience: if the single compartment had been experienced before, tuning curves would revert to their previous unimodality. If not, it is likely that ENV and CONJ cells would initially remain bidirectional when returning to a single compartment, as both cell types would be still driven by two separate HDs. However, there remains the possibility that over longer exposures they will learn this new environment well and become unidirectional.

What would happen if the rat only experienced a single-compartment environment having two fold rotational symmetry? Our model would suggest that we would also see BC cells, which would not be distinguishable from WC cells (the cells would show bipolar tuning curves) except that the peaks would be symmetrical. However, it may be that in reality, something about the transition from one compartment to another enabled disengagement of BC cells from the HD signal.

How dependent is the system on the HD inputs? We do not yet have experimental data on this issue, but one possibility is that if the HD inputs were inactivated, perhaps optogenetically, then BC cells would continue to show directionality because these are being driven by the visual inputs. Meanwhile, WC cells would resemble BC cells, with only a single tuning curve that reversed. It is possible however that the BC pattern is itself dependent on an HD input, at least to establish the tuning curve in the first place. Thus, while the model predicts persistence, this is a matter for empirical testing.

We did not model the development of the VIS inputs between sensory cortex and RSC, instead wiring these by hand; this would be a natural extension to the model. We also did not model the contextual inputs to the system, instead breaking the symmetry by hand by allocating HD cells to be North vs. South etc. In reality, the HD system itself would only be able to orient stably from trial to trial by incorporating the olfactory cues, which enable the animal to distinguish North from South. Without these, experimentally we may have seen random orientation of the HD cells from one trial to the next, with consequent reversal of the whole HD-BC-WC pattern. However, it seems certain that the pattern is dependent on contextual cues in addition to the visual landmarks. Future work, both experimental and theoretical, therefore could explore the role of the feedback inputs from hippocampus (mainly via subiculum) in establishing the firing patterns.

The 180° conflict between global HD and the local visual environment is a specific case of cue conflict more generally. If RSC mediates environmental directional influence on the HD system, then we can also predict CONJ and ENV behavior during cue conflict resolution such as that seen by Knight et al. (2013), in which cells performed weighted integration of their inputs. In this experiment, rats were exposed to a change in direction of a single landmark in a circular environment, and HD cells were observed to rotate to a point between the original direction and that indicated by the new landmark direction. A next step for our model might be to see whether it can resolve this cue conflict in the same manner as we suggested in our previous theoretical work (Page et al., 2014), which proposed rapid re-weighting of the visual inputs as a mechanism for the cue integration. Importantly, our simulations predict specific patterns of conjunctive cell activity during cue conflict situations: ENV cells should have unimodal tuning curves with a peak at the direction indicated by visual input, CONJ cells should have bimodal tuning curves with a peak each at the internal and visual input directions. The separation in CONJ cell tuning curve peaks should dynamically reflect the separation between directions signaled by the ADN HD cells, and by the VIS inputs. As cue conflict is resolved, this separation should be reduced.

While the present model does not directly replicate the finding of Jacob et al. (2017) that bidirectional cells remain bidirectional in the dark, it should be noted that, as per point 6 in “Model Description and Simulation” section, the VIS cell representation would likely in reality be replaced with more multimodal (especially tactile or olfactory) inputs, in which case bidirectional cell tuning properties would be preserved in the dark.

### Function of This Network

Why should the brain need BC and WC cells as well as HD cells? In other words, why should a two-way interaction exist between the HD signal and the incoming sensory inputs? We have proposed here that this arrangement helps the brain solve two landmark-processing problems, the proximal-distal problem and the landmark stability problem. These problems relate to the perceptual stability of landmarks. Both landmark movement and directional inconsistency are deleterious for directional orientation, and result in disconnection of landmarks from the spatial representation. In the case of landmark instability, place and HD cells cease rotating their activity in response to rotations of landmarks that move (Knierim et al., 1995; Jeffery, 1998). In the case of directional inconsistency, HD and place cells rotate their firing to follow distant (distal) directional cues (Zugaro et al., 2001; Yoganarasimha et al., 2006) but not if the cues are so nearby (proximal) that the animal can walk around them (Cressant et al., 1997).

Our hypothesis is that RSC solves these problems by linking its current perceptual inputs (the environmental landmark array, called in our model the LSE) with the HD signal, and then using Hebbian coincidence detection to resolve any mismatch between expected and actual perception arising from either instability of the HD signal or instability of the environmental landmarks. The BC cells have the function of tracking landmarks, while HD cells, which are organized in the subcortical attractor networks, reflect the brain’s current estimate of facing direction. Repeated congruence between the LSE/BC cells and the HD signal would cause Hebbian strengthening of links to the HD system, such that these cells would come to reset the HD signal should it drift. In a stable environment, then, BC cells would look like HD cells, which may be why they have not been seen previously. Repeated incongruence, either because landmarks are proximal or because they are actually unstable, would cause a disjunction in LSE/BC and HD activity, with consequent weakening of the links.

We simulated this proposal with a simple environment having 12, identical, evenly arranged distal cues. We chose these parameters for simplicity, as this was a proof-of-principle experiment, but in reality the inputs from the visual system would be far richer in their content, which ought to make precision of HD encoding even easier. In reality it is very likely that the brain samples a set of visual snapshots, each subtending some considerable range of the visual field. It is possible therefore that the visual inputs as the rat turns to face different directions comprise thousands of such snapshots, each slightly different. However, our simulation demonstrated that even in a visually impoverished environment, the statistics of landmark visibility allow the system to use distal landmarks to orient HD cells without location-dependent distortion (parallax error), making this HD-BD interaction a potentially powerful mechanism for stabilizing the signal.

This mechanism works for distal but not proximal landmarks because distal landmarks occur relative to HD with a peaked probability distribution, whilst centrally-placed landmarks do not (Figure 12B). This peaked probability distribution means that a CONJ or ENV cell driven by VIS is active most often at a given HD, and thus a peaked Hebbian association could be made with specific HD cells. This is turn reinforces the relationship between VIS activity and these CONJ/ENV cells. We demonstrated here that whilst this is the case for distal landmarks, which can stabilize the HD signal once learned, centrally-placed proximal landmarks, by virtue of their uniformly distributed probabilities of occurrence relative to HD, fail to induce specific connectivity and this directly affects HD signal stability. Such a mechanism could account for the fact that distal landmarks are used preferentially by the HD system (Cressant et al., 1997; Zugaro et al., 2001), since proximal landmarks will have broader probability distributions relative to HD.

To summarize: a theoretical mechanism is presented for the phenomenon of bidirectional cells seen by Jacob et al. (2017). This mechanism explains key properties of bidirectional cells including WC (CONJ) and BC (ENV) directionality, presence and BC flipping of dominant peaks in CONJ tuning curves, the persistent unidirectionality of HD cells, loss of tuning curve specificity in the open field, and the persistence of bidirectionality in the dark. The model yields a number of testable predictions, and suggests a reason for the HD system affinity for distal landmarks of proximal/intramaze landmarks, via a mechanism by which the parallax problem might also be solved.

## Author Contributions

HP and KJ devised the model together and wrote the manuscript. HP constructed and tested the model.

## Conflict of Interest Statement

The authors declare that the research was conducted in the absence of any commercial or financial relationships that could be construed as a potential conflict of interest.
