# Supplementary material for: Landmark-Based Updating of the Head Direction System by Retrosplenial Cortex: A Computational Model
Source: Front Cell Neurosci. 2018 Jul 13;12:191. doi: 10.3389/fncel.2018.00191 (PMC6055052; doi:10.3389/fncel.2018.00191)
Supplement: Supplementary file 1 [file Data_Sheet_1.DOCX]

**Supplementary Material**

**Model Equations**

***RSC HD Cells***

The RSC HD layer is a ring of cells, with each cell *i* having a preferred firing direction *xi* distributed in the range 0-360◦. Importantly, these cells do not have direct excitatory influence on one another. Cells are arranged topographically, such that adjacent HD cells have adjacent preferred firing directions. This arrangement is for each of visualisation and analysis and does not reflect HD cell organisation in the rat brain, where no such topography has been observed. In this manner a contiguous Gaussian packet of activity in the HD layer represents current head direction. Both types of connectivity contain a conduction delay Δt.

Cells are implemented using a rate coded leaky-integrator neuronal model. The activation level $h_{i}^{HD}(t)$ of a given HD cell *i* at time *t* is given by

$$\tau^{HD}\frac{dh_{i}^{HD}\left( t \right)}{dt}= - h_{i}^{HD}\left( t \right)$$

$$+ \frac{\varphi_{4}}{C^{ADN\to HD}}\sum_{j} w_{ij}^{4}r_{j}^{ADN}\left( t-\Delta\right)$$

$$-\frac{1}{N^{HD}}\sum_{j} \tilde{w}^{HD}r_{j}^{HD}\left( t \right)+\frac{\varphi_{2}}{C^{CONJ+ENV\to HD}}\sum_{j} w_{ij}^{2}r_{j}^{WC+BC}(t-\Delta)$$

The term $- h_{i}^{HD}\left( t \right)$ reflects exponential decay, whereby in the absence of any input, activation will fall to zero at a rate determined by the time constant τ. The term $\frac{1}{N^{HD}}\sum_{j} \tilde{w}^{HD}r_{j}^{HD}\left( t \right)$ represents within-layer inhibitory feedback, described by $\tilde{w}^{HD}$, a global constant approximating the effect of inhibitory interneurons. This term is summed over all presynaptic HD cells *j*, and scaled by the number of cells in the layer $N^{HD}.$

Presynaptic input from CONJ and ENV cells is reflected by the term $\frac{\varphi_{2}}{C^{CONJ+ENV\to HD}}\sum_{j} w_{ij}^{2}r_{j}^{WC+BC}(t-\Delta)$, where $w_{ij}^{2}$ is the weight from presynaptic CONJ/ENV cell *j* with rate $r_{j}^{WC+BC}$ to postsynaptic HD cell *i*. It is summed over all presynaptic cells, and scaled by both the constant $\varphi_{2}$and the number of connections *i* receives, $C^{CONJ+ENV\to HD}$.

In an analogous manner, the term $+ \frac{\varphi_{4}}{C^{ADN\to HD}}\sum_{j} w_{ij}^{4}r_{j}^{ADN}\left( t-\Delta\right)$ reflects presynaptic input from ADN cells.

***ADN HD Cells***

The ring of ADN cells provides ascending HD input to RSC via w^4^ connections. Similarly to the activation level of HD cells, the activation level $h_{i}^{ADN}(t)$ of a given ADN cell *i* at time *t* is given by

$$\tau^{ADN}\frac{dh_{i}^{ADN}\left( t \right)}{dt}= - h_{i}^{ADN}\left( t \right)$$

$$+ \frac{\varphi_{6}}{C^{ADN\to ADN}}\sum_{j} w_{ij}^{6}r_{j}^{ADN}\left( t-\Delta\right)$$

$$+ \frac{\varphi_{5}}{C^{HD\to ADN}}\sum_{j} w_{ij}^{5}r_{j}^{HD}\left( t-\Delta\right)$$

$$-\frac{1}{N^{ADN}}\sum_{j} \tilde{w}^{ADN}r_{j}^{ADN}\left( t \right)$$

Note that the ADN layer projects back to itself, acting as a recurrent neural network. It also differs from the HD layer in that it is driven by path integration input represented by the term $e_{i}\left( t \right)$. This is calculated according to the Gaussian function

$$e_{i}=\lambda^{PI}e^{{-{(s_{i}^{PI})}^{2}}/{{2(\sigma^{PI})}^{2}}}$$

where $\lambda^{PI}$ is a scaling parameter and $\sigma^{PI}$is the standard deviation of the Gaussian profile. $s_{i}^{PI}$ reflects the difference between *x_i_* the PFD of cell *i* and the current direction as estimated by path integration, *x^est^*. In order to create a wraparound effect in circular space, it is calculated as

$$s_{i}^{PI}=\min_{} \left( \left| x_{i}-x^{est} \right|,360-\left| x_{i}-x^{est} \right| \right)$$

The overall effect is to generate a Gaussian input to the ADN layer centred on *x^est^*. In order to simulate noisy path integration, *x^est^* is initially set to the true HD of the animal, but at each subsequent timestep it is updated by the change in true HD $\theta$ plus a noise term $\gamma$

$$x^{est}\left( t \right)=x^{est}\left( t-1 \right)+\min_{} \left( \left| \theta(t)-\theta(t-1) \right|,360-\left| \theta(t)-\theta(t-1) \right| \right)+\gamma$$

The noise term is drawn from a random normal distribution, centred on a value set as a parameterised percentage of the change in HD, ξ, and with a range also set as a percentage of this change, $\sigma^{NOISE}$.

***CONJ and ENV Cells***

Cells in the CONJ/ENV layer both receive *w^3^* connections from the VIS layer, and both project back onto the HD layer with *w^2^* connections. However, only CONJ cells receive *w^1^* connections from the HD layer. The activation level of $h_{i}^{CONJ}\left( t \right)$ of CONJ cell *i* is given by

$$\tau^{CONJ/ENV}\frac{dh_{i}^{CONJ}\left( t \right)}{dt}= - h_{i}^{CONJ}\left( t \right)-\frac{1}{N^{CONJ/ENV}}\sum_{j} \tilde{w}^{CONJ/ENV}r_{j}^{CONJ/ENV}\left( t \right)+\frac{\varphi_{1}}{C^{HD\to CONJ}}\sum_{j} w_{ij}^{1}r_{j}^{HD}(t-\Delta)+\frac{\varphi_{3}}{C^{VIS\to CONJ/ENV}}\sum_{j} w_{ij}^{3}r_{j}^{VIS}(t)$$

these terms are analogous to those used to calculate $- h_{i}^{HD}$, with *VIS* denoting values relating to VIS cells, *CONJ* denoting CONJ cells, and *CONJ/ENV* denoting either CONJ or ENV cells.

The activation of ENV cells is given similarly to the above, with the omission of any input terms relating to HD cells.

***Transfer function***

The firing rate $r_{i}\left( t \right)$ of HD, ADN, CONJ, and ENV cells *i* at time *t* is calculated as a hyperbolic tangent function of the activation level $h\left( t \right)$. This function is naturally bound in the interval [-1, 1]. However, real neurons cannot have firing rates below 0, so the final transfer function is bounded in the form

$$r_{i}\left( t \right)=\left\{ \begin{aligned} 0, &\tanh(h_{i}(t))<0 \\ \tanh(h_{i}(t)), &\tanh(h_{i}(t))\geq0 \end{aligned} \right.$$

***VIS Cells***

The VIS layer consists of cells that represent directionality in terms of the local spatial environment. These cells are theorised be principally visual, although in reality can reflect a number of local allothetic sources of directional information. VIS cells have preferred firing directions in the range 0-360◦, and can represent direction as a Gaussian packet of activity similarly to the RSC HD layer. However, VIS cells signal head direction only in reference to the current local environment. The location of a VIS activity packet in these simulations will therefore shift 180° between compartments in the two-compartment apparatus, and by 120◦ in the three-compartment apparatus. The firing of VIS cells is determined in a very similar manner to ADN input, but the rates of VIS cells are set directly rather than having input applied to them. Their rates are set as

$$r_{i}^{VIS}=\lambda^{VIS}e^{{-{(s_{i}^{VIS})}^{2}}/{{2(\sigma^{VIS})}^{2}}}$$

In this case, $s_{i}^{VIS}$ reflects the difference between the preferred firing direction of a given postsynaptic VIS cell, $x_{i}^{VIS}$, and $\theta^{VIS}$ the true head direction *with reference to local space*

$$r_{i}^{VIS}=\min_{} \left( \left| x_{i}^{VIS}-\theta^{VIS} \right|,360-\left| x_{i}^{VIS}-\theta^{VIS} \right| \right)$$

Crucially, $\theta^{VIS}$varies relative to the true HD of the animal θ between compartments based on

$$\theta^{VIS}=\left\{ \begin{aligned} \theta, &compartment=1 \\ \theta+180, compartment=2 \end{aligned} \right.$$

for two-compartment simulations, and for three-compartment simulations

$$\theta^{VIS}=\left\{ \begin{aligned} \theta, &compartment=1 \\ \theta+120, compartment=2 \\ \theta-120, compartment=3 \end{aligned} \right.$$

In a modified version of the model, VIS cells are altered to act as binary landmark detectors, firing at a rate of 1 if a landmark falls within a 90^o^ field of view and at 0 otherwise.

For each landmark *i*, and therefore each VIS cell, the process is as follows. At each simulation timestep, a vector is constructed from the rat’s current position to a given landmark

$$\boldsymbol{L}\boldsymbol{V}_{\boldsymbol{i}}\boldsymbol{=(}\boldsymbol{x}_{\boldsymbol{i}}^{\boldsymbol{LM}}\boldsymbol{-posx}\left( \boldsymbol{t} \right)\boldsymbol{,}\boldsymbol{y}_{\boldsymbol{i}}^{\boldsymbol{LM}}\boldsymbol{-posy}\left( \boldsymbol{t} \right)\boldsymbol{)}$$

The non-signed angle between a normalized version of this vector, and the current heading vector is calculated as

$$\boldsymbol{\alpha}_{\boldsymbol{i}}\boldsymbol{(t)= acos(L}\boldsymbol{V}_{\boldsymbol{i}}\boldsymbol{\cdot HD(t))}$$

Finally, the rate of the vis cell corresponding to this landmark is set as

$${r_{i}}^{VIS}\left( t \right)=\left\{ \begin{aligned} 1, &\alpha_{i}(t)<FOV/2 \\ 0, &otherwise \end{aligned} \right.$$

where FOV is the width of the rat’s field of view (set to 90^o^ for the simulations presented in this paper).

***Self-organising network weights***

Both HD to CONJ and CONJ/ENV to HD weights self-organise over the course of simulation. In cue conflict simulations, CONJ/ENV to HD weights are pre-wired, but update during simulation. In landmark learning simulations, VIS to CONJ/ENV weights also self-organise. Weight update rules take the same form regardless of which set of weights they are operating upon

$$\frac{dw_{ij}(t)}{dt}=kr_{i}\left( t \right)r_{j}(t-\Delta t)$$

where *k* is the learning rate parameter. All weights are normalised after updating. This is achieved by ensuring that the square root of the sum of the squares of all the presynaptic weights for each given postsynaptic cell is limited to 1. It is achieved by

$$\sqrt{\sum_{j} {{(w}_{ij}(t))}^{2}}=1$$

***Pre-wired connectivity***

Connectivity from VIS cells to CONJ and ENV cells (*w^3^*), from ADN to ADN (*w^6^*), from ADN to HD (*w^4^*) and from HD to ADN (*w^5^*) is pre-wired, and does not change over the course of simulation, with the exception of VIS to CONJ/ENV weights in landmark learning simulations. Preferred firing directions in the range 0-360 are assigned to CONJ cells and ENV cells independently, such that each population of cells covers the entire range. Connectivity from presynaptic VIS cell *j* to postsynaptic CONJ/ENV cell *i* is given by another Gaussian in the form

$$w_{ij}^{3}=e^{-{{(s_{ij}^{VIS})}^{2}}/{2{(\sigma^{3})}^{2}}}$$

where $\sigma^{3}$ is the standard deviation, and $s_{ij}^{LSE}$ is the difference between the preferred firing direction of VIS cell *j* and of CONJ/ENV cell *i.* It is calculated with a wraparound effect, analogously to wraparounds already used in the methods.

**Forward Euler methodology**

The differential equations given above cannot be solved analytically. They are implemented in the computer model by making discrete approximations of their solutions using a Forward Euler finite difference scheme with time step δ*t*. By way of illustration, consider a simplified version of an equation calculating the activation level of a single HD cell

$$\tau^{HD}\frac{dh_{i}^{HD}\left( t \right)}{dt}= - h_{i}^{HD}\left( t \right)+ f_{i}(t)$$

Using the Forward Euler method, this is implemented in the form

$$h_{i}^{HD}\left( t+\delta t \right)=h_{i}^{HD}\left( t \right) - {\frac{\delta t}{\tau^{HD}}h}_{i}^{HD}\left( t \right)+ \frac{\delta t}{\tau^{HD}}f_{i}(t)$$

All activation and synaptic weight updates are implemented in the model in this manner.

**Model parameters used**

***Two-compartment simulations:***

| Simulation Time | 600 s |
| --- | --- |
| Timestep Size (δ*t*) | 0.0001s |
| RSC HD Cells ($N^{HD}$) | 180 |
| ADN HD Cells ($N^{ADN}$) | 180 |
| Bidirectional Cells ($N^{CONJ/ENV}$) | 360 |
| VIS$cells (N^{VIS}$) | 180 |
| HD to Bidirectional Strength ($\varphi_{1}$) | 160 |
| Bidirectional to HD Strength ($\varphi_{2}$) | 45 |
| VIS to Bidirectional Strength ($\varphi_{3}$) | 100 |
| ADN to RSC HD Strength ($\varphi_{4}$) | 120 |
| RSC HD to ADN Strength ($\varphi_{5}$) | 10 |
| ADN to ADN Strength ($\varphi_{6}$) | 40 |
| RSC HD Time Constant ($\tau^{HD}$) | 0.001s |
| Bidirectional Time Constant ($\tau^{CONJ/ENV}$) | 0.001s |
| ADN Time Constant ($\tau^{ADN}$) | 0.001s |
| HD Inhibition ($\tilde{w}^{HD}$) | 20 |
| Bidirectional Inhibition ($\tilde{w}^{CONJ/ENV}$) | 7 |
| ADN Inhibition ($\tilde{w}^{ADN}$) | 5 |
| Conduction Delay (Δt) | 0.001 |
| Learning Rate (k) | 0.005 |
| HD to Bidirectional Weight Width ($\sigma^{1}$) | 20 |
| Bidirectional to HD Weight Width ($\sigma^{2}$) | 20 |
| VIS to Bidirectional Weight Width ($\sigma^{3}$) | 20 |
| ADN to HD Weight Width ($\sigma^{4}$) | 10 |
| HD to ADN Weight Width ($\sigma^{5}$) | 10 |
| ADN to ADN Weight Width ($\sigma^{6}$) | 10 |
| VIS Input Width ($\sigma^{VIS}$) | 10 |
| VIS Input Strength ($\lambda^{VIS}$) | 4 |
| Path Integration Input Width ($\sigma^{PI}$) | 10 |
| PI Noise Width ($\sigma^{NOISE}$) | 0.5% |
| PI Noise Amount (ξ) | 0.5% |
| PI Input Strength ($\lambda^{PI}$) | 4 |

***Three-compartment simulations:***

| Simulation Time | 600 s |
| --- | --- |
| Timestep Size (δ*t*) | 0.0001s |
| RSC HD Cells ($N^{HD}$) | 180 |
| ADN HD Cells ($N^{ADN}$) | 180 |
| Bidirectional Cells ($N^{CONJ/ENV}$) | 360 |
| VIS$cells (N^{VIS}$) | 180 |
| HD to Bidirectional Strength ($\varphi_{1}$) | 90 |
| Bidirectional to HD Strength ($\varphi_{2}$) | 45 |
| VIS to Bidirectional Strength ($\varphi_{3}$) | 100 |
| ADN to RSC HD Strength ($\varphi_{4}$) | 120 |
| RSC HD to ADN Strength ($\varphi_{5}$) | 10 |
| ADN to ADN Strength ($\varphi_{6}$) | 40 |
| RSC HD Time Constant ($\tau^{HD}$) | 0.001s |
| Bidirectional Time Constant ($\tau^{CONJ/ENV}$) | 0.001s |
| ADN Time Constant ($\tau^{ADN}$) | 0.001s |
| HD Inhibition ($\tilde{w}^{HD}$) | 20 |
| Bidirectional Inhibition ($\tilde{w}^{CONJ/ENV}$) | 2 |
| ADN Inhibition ($\tilde{w}^{ADN}$) | 5 |
| Conduction Delay (Δt) | 0.001 |
| Learning Rate (k) | 0.01 |
| HD to Bidirectional Weight Width ($\sigma^{1}$) | 5 |
| Bidirectional to HD Weight Width ($\sigma^{2}$) | 20 |
| VIS to Bidirectional Weight Width ($\sigma^{3}$) | 5 |
| ADN to HD Weight Width ($\sigma^{4}$) | 10 |
| HD to ADN Weight Width ($\sigma^{5}$) | 10 |
| ADN to ADN Weight Width ($\sigma^{6}$) | 10 |
| VIS Input Width ($\sigma^{VIS}$) | 20 |
| VIS Input Strength ($\lambda^{VIS}$) | 4 |
| Path Integration Input Width ($\sigma^{PI}$) | 10 |
| PI Noise Width ($\sigma^{NOISE}$) | 0.5% |
| PI Noise Amount (ξ) | 0.5% |
| PI Input Strength ($\lambda^{PI}$) | 4 |

***Landmark learning simulations:***

| Simulation Time | 600 s |
| --- | --- |
| Timestep Size (δ*t*) | 0.0001s |
| RSC HD Cells ($N^{HD}$) | 180 |
| ADN HD Cells ($N^{ADN}$) | 180 |
| Bidirectional Cells ($N^{CONJ/ENV}$) | 360 |
| VIS$cells (N^{VIS}$) | 12 |
| HD to Bidirectional Strength ($\varphi_{1}$) | 60 |
| Bidirectional to HD Strength ($\varphi_{2}$) | 45 |
| VIS to Bidirectional Strength ($\varphi_{3}$) | 15 |
| ADN to RSC HD Strength ($\varphi_{4}$) | 120 |
| RSC HD to ADN Strength ($\varphi_{5}$) | 10 |
| ADN to ADN Strength ($\varphi_{6}$) | 40 |
| RSC HD Time Constant ($\tau^{HD}$) | 0.001s |
| Bidirectional Time Constant ($\tau^{CONJ/ENV}$) | 0.001s |
| ADN Time Constant ($\tau^{ADN}$) | 0.001s |
| HD Inhibition ($\tilde{w}^{HD}$) | 20 |
| Bidirectional Inhibition ($\tilde{w}^{CONJ/ENV}$) | 10 |
| ADN Inhibition ($\tilde{w}^{ADN}$) | 5 |
| Conduction Delay (Δt) | 0.001 |
| Learning Rate (k) | 0.01 |
| HD to Bidirectional Weight Width ($\sigma^{1}$) | 20 |
| Bidirectional to HD Weight Width ($\sigma^{2}$) | 20 |
| VIS to Bidirectional Weight Width ($\sigma^{3}$) | 20 |
| ADN to HD Weight Width ($\sigma^{4}$) | 10 |
| HD to ADN Weight Width ($\sigma^{5}$) | 10 |
| ADN to ADN Weight Width ($\sigma^{6}$) | 10 |
| VIS Input Width ($\sigma^{VIS}$) | N/A |
| VIS Input Strength ($\lambda^{VIS}$) | N/A |
| Path Integration Input Width ($\sigma^{PI}$) | 10 |
| PI Noise Width ($\sigma^{NOISE}$) | 6% |
| PI Noise Amount (ξ) | 0.5% |
| PI Input Strength ($\lambda^{PI}$) | 4 |
